# Evaluation of uranium migration during the maturation of hydrocarbon source rocks

**DOI:** 10.1038/s41598-024-75930-z

**Published:** 2024-11-06

**Authors:** Junxian Wang, Ziying Li, Feng He, Fengtian Bai, Linfei Qiu, Jian Guo, Chuang Zhang

**Affiliations:** 1 Beijing Research Institute of Uranium Geology, CNNC, No. 10 Xiaoguandongli, Chaoyang District, Beijing, 100029 China; 2 National Key Laboratory of Uranium Resource Exploration-Mining and Nuclear Remote Sensing, Beijing, 100029 China; 3 College of Construction Engineering, Jilin University, Changchun, 130061 Jilin China

**Keywords:** Uranium, Black shale, Source rock, Organic acid, Element migration, Solid Earth sciences, Sedimentology

## Abstract

The source of uranium is an important research topic related to the exploration of sandstone-type uranium deposits, and potential uranium sources in deep basins are often overlooked. Black organic-rich shale is a common uranium-bearing rock in deep sedimentary basins. However, relatively few studies have investigated the migration of uranium during hydrocarbon generation in and release from uranium-rich shale. In this study, the uranium-rich shale in the Chang 7 member of the Yanchang Formation of the Upper Triassic in the Ordos Basin was selected to investigate the migration of uranium and other trace elements during the thermal maturation of uranium-rich shale via a semiopen pyrolysis simulation system. The gas and liquid products as well as the solid residue were thoroughly analysed by means of multiple instruments. The results showed that uranium significantly migrated before hydrocarbon generation (Ro < 0.61%), with a leaching rate between 12.1% and 18.8%. The leaching rate of uranium during the hydrocarbon generation stage (0.63% < Ro < 1.35%) was relatively low, ranging from 0 to 7.2%. Cu, Pb, Zn, Mo, and other trace elements also migrated considerably during the early stage of thermal evolution, with leaching rates ranging from 2.9 ~ 11.6%. The yield of low-molecular-weight organic acids (LOAs) was the highest in the early stage of thermal maturity, and the LOA yield exhibited a good correlation with the leaching rates of Cu, Pb, Zn, Co, Mo, etc. The generation of LOAs from source rocks was conducive to the leaching and migration of trace elements. Moreover, according to a statistical analysis of published geochemical data, the total organic carbon (TOC) content, uranium content, and U/TOC ratio in shale decreased significantly with increasing burial depth, indicating that uranium migrated significantly upon kerogen hydrocarbon generation during thermal evolution. Therefore, uranium-rich shale is an important deep uranium source in sedimentary basins.

**Supplementary Information:**

The online version contains supplementary material available at 10.1038/s41598-024-75930-z.

## Introduction

With the rapid development of the global nuclear power industry, the demand for uranium resources has increased rapidly, and the exploration of uranium resources needs to expand in scope. During uranium exploration, the source of ore-forming elements is an important factor, especially during sandstone-type uranium exploration, and determining whether a granite weathering area exists at the edge of the basin that provides sufficient uranium is important for selecting exploration targets^1–3^. The most recent research results show that, in addition to traditional oxidation-reduction-based genesis, the exudation type uranium mineralization induced by uranium-rich fluid derived from deep uranium-rich formations in basins hinterland is also an important metallogenesis process^4–7^. Therefore, the source potential of uranium-rich shale in the deep part of the basin cannot be ignored.

Organic-rich black shale, such as the Alum shale of the Lower Palaeozoic in northern Europe^8,9^, the Upper Carboniferous Gastrioceras Listeri marine shale in England^10^, the Upper Devonian New Albany Shale in the Illinois Basin in the eastern United States^11^, the Cambrian Niutitang Formation marine shale in southern China^12^, and the lacustrine shale of the Triassic Yanchang Formation in the Ordos Basin in northern China^13^, often has a high uranium content. These uranium-rich shales were formed in anoxic to reductive palaeowaters with abundant organic matter^14,15^. Due to the high cost of hydrometallurgy, uranium in shale is usually difficult to use and is often used as a harmful associated element during oil shale retorting to produce heavy oil^16,17^.

Many studies have shown that black shale rich in trace elements can provide sufficient metal elements for mineralization in its upper part during diagenetic evolution^18–20^, and several important MVT-type lead-zinc ore-forming elements have been confirmed to be derived mainly from black shale^21^. Previous simulations have confirmed that Zn, U, Au and other metals have high solubility in hydrocarbons, so hydrocarbons are potential ore-forming fluids^22,23^. Especially in the early stage of thermal evolution of sedimentary organic matter, many low-molecular-weight organic acids (LOAs) are generated, and these acids can readily corrode minerals^24–27^ and form complexes with several trace metal elements, thus improving the migration ability of these elements^28–30^. However, at present, experimental research on the leaching of trace metal elements during thermal maturation of sedimentary organic matter is scarce.

Therefore, for this study, the uranium-rich shale in the Chang 7 member of the Yanchang Formation of the Upper Triassic in the Ordos Basin was selected as the experimental subjects to determine the migration of uranium and other trace elements during the thermal maturation of uranium-rich shale. A semiopen pyrolysis simulation system was applied to simulate the generation of solid and liquid products in different maturation stages, and the fluid compositions and element migration patterns in different stages were analysed. Finally, published geochemical data from shale samples at different depths in the same layer in the whole basin were statistically analysed to further determine the uranium content and relative abundance of organic matter at different burial depths (maturity stages) to investigate the migration of uranium under natural geological conditions. This study is expected to contribute to the evaluation of the potential of deep uranium sources during exudation-type uranium mineralization and to be highly important for improving the understanding of sandstone-type uranium mineralization.

### Geological setting

**Fig. 1:**
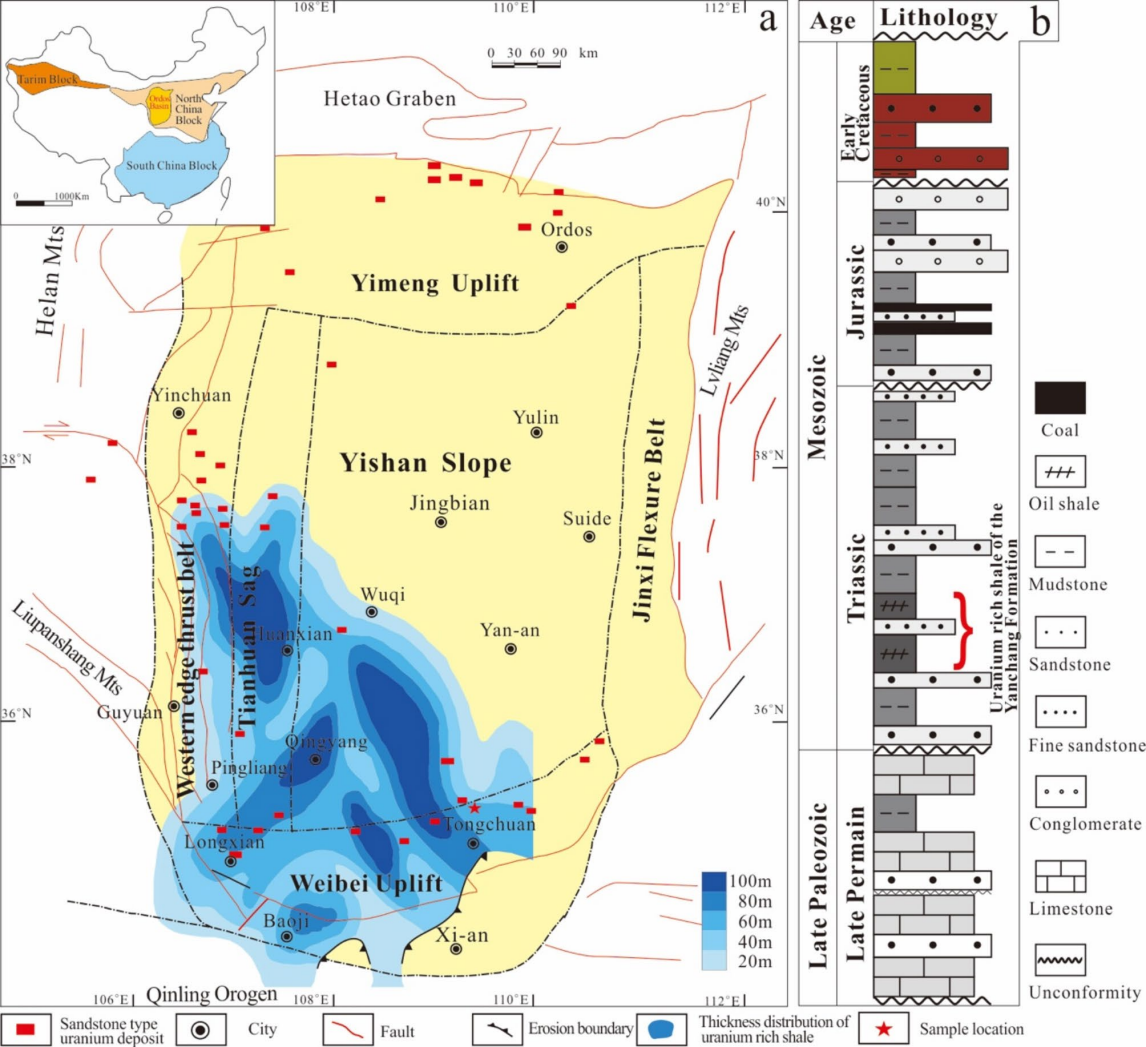
(**a**) Location, structural zoning, and distribution map of uranium-rich shale (Chang 7 member of the Yanchang Formation) and uranium deposits in the Ordos Basin^32,39^ and (**b**) stratigraphic column of Mesozoic rocks in the Ordos Basin. Created by CorelDRAW Graphics Suite 2024 (https://www.coreldraw.com/cn/product/coreldraw/#).

The Ordos Basin is a large continental sedimentary basin developed from the Palaeozoic to the Mesozoic in Central China. The structural form of the basin is an asymmetric syncline with a north-south trend, with a gentle slope in the east and a steep slope in the west. It is approximately 400 km wide from east to west and 640 km long from south to north and covers an area of approximately 25 × 10^4^ km^2^. According to the internal structural characteristics, the basin can be divided into six secondary structural units, namely, the Yimeng uplift, the Weibei uplift, etc. (Fig. 1a)^31,32^. The sedimentary cover of the basin is mainly composed of Mesozoic and Cenozoic terrigenous clastic rocks. The Triassic, Jurassic and Lower Cretaceous strata are the main deposits in the basin. The Mesozoic internal strata rise in the east and fall in the west^33,34^. The Triassic Yanchang Formation shale is the source rock of almost all the oil and a portion of the natural gas in the basin (Fig. 1b). It is widely distributed in the southern part of the basin, with a cumulative thickness of up to 100 m, and most of the shale are excellent hydrocarbon source rocks^35,36^. The most significant feature of the Yanchang Formation shale is its high content of uranium, with an average value of 32.8 ppm^37^.

Many sandstone-type uranium deposits have been found in the southern and northern regions of the basin^38,39^. Notably, there are significant differences in metallogenic characteristics between sandstone-type uranium deposits in the northern and southern parts of the basin. Uranium deposits in the northern part of the basin are mainly of redox origin, and their occurrence is controlled by the redox transition zone^39,40^. The uranium deposits in the southern region have obvious vertical superimposed relationships with the uranium-rich shale in the deep regions of the Chang 7 member of the Triassic Yanchang Formation. Many uranium ore bodies occur in aeolian sand deposits that lack reducing substances, and even thick industrial ore bodies are found in large-scale primary red oxide layers^41^. The orebody horizon is closely related to strike-slip faults and has characteristics typical of exudation-type uranium mineralization^4^. The differences in the characteristics and metallogenic types between the northern and southern parts of the basin indicate that uranium has different sources during metallogenesis; that is, in the southern part of the basin, the Triassic Yanchang Formation under the sandstone-type uranium deposits of the Lower Cretaceous Huanhe Formation is a potential deep uranium source rock.

### Samples and methods

To determine the migration characteristics of elements at different maturity stages, the major and trace elements in solid residual samples after pyrolysis at different temperatures were analyzed to determine the element leaching rate. Moreover, the yield of hydrocarbons and the composition of LOAs in the fluid products at different stages were determined to evaluate the effect of the characteristics of fluid products on element leaching.

### Samples

**Fig. 2:**
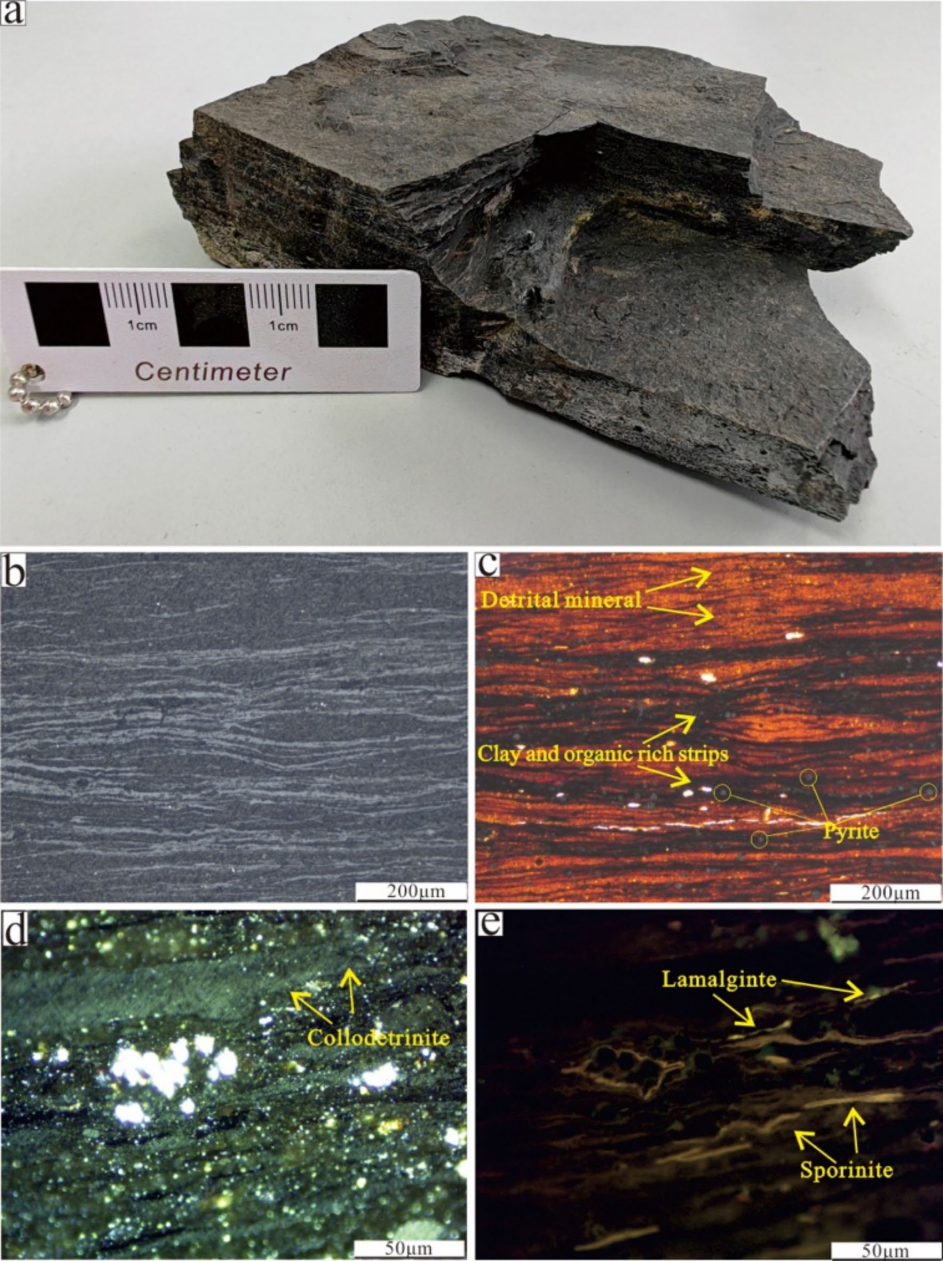
(**a**) Macroscopic characteristics of shale samples, (**b**) microscopic mineralogical characteristics (orthogonal polarization), (**c**) microscopic mineralogical characteristics (single polarization), (**d**) maceral characteristics (white light), and (**e**) maceral characteristics (fluorescence).

The shale samples from member 7 of Triassic Yanchang Formation was taken from an open pit of oil shale in the northern region of Tongchuan city in the southern Ordos Basin (Fig. 1a). The sample was a grey black block with horizontal bedding (Fig. 2a), and terrigenous clastic minerals such as quartz and feldspar were interbedded with organic-rich clay in laminar form (Fig. 2b, c). The organic macerals in the shale were mainly algae, followed by terrigenous crustaceous components such as sporophytes and vitrinite (Fig. 2d, e). Before the experiment, the large sample was crushed and passed through screens to collect 10–60 mesh particles, and the sample was made fully uniform by stirring.

**Fig. 3:**
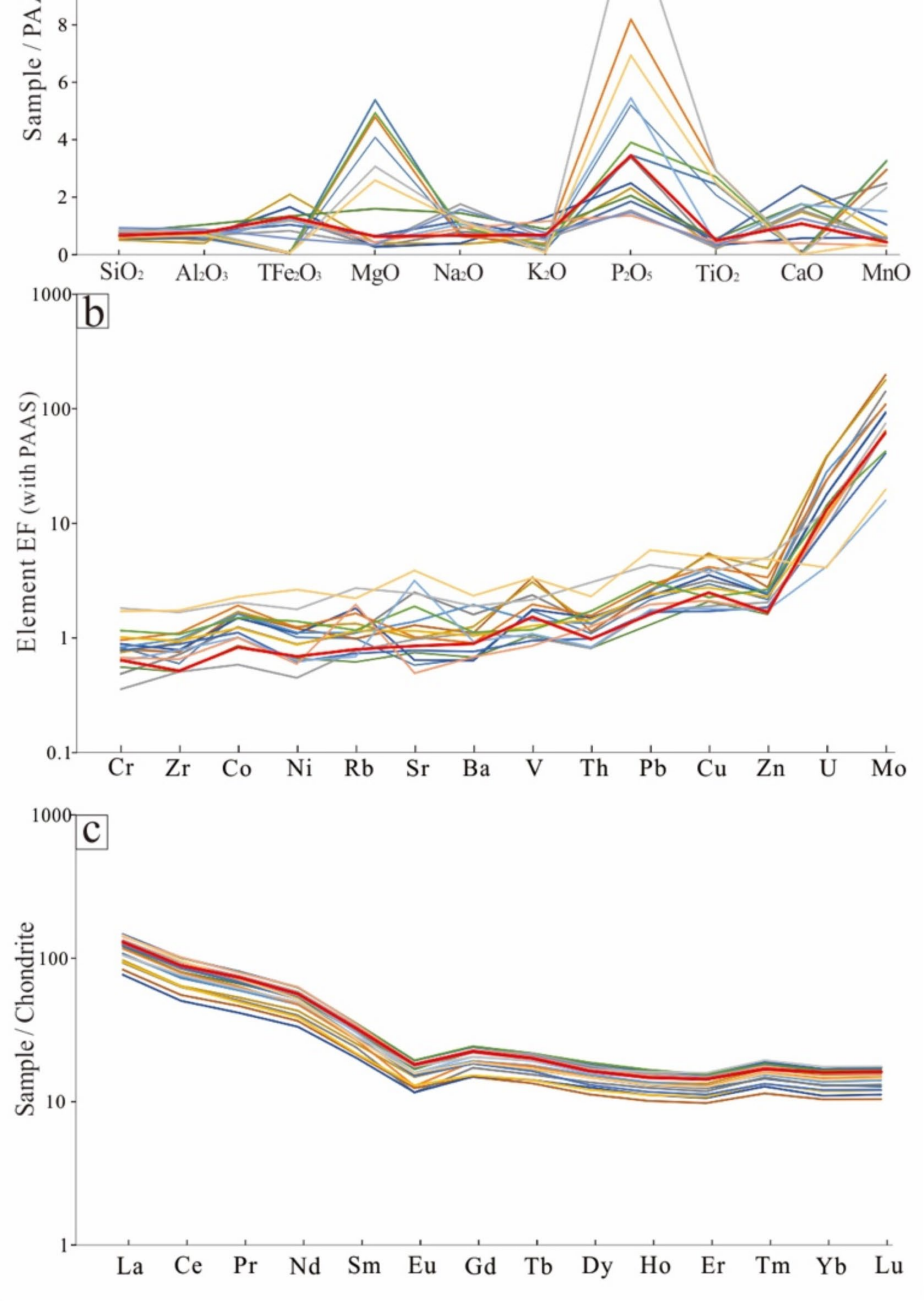
Geochemical characteristics of the experimental samples and Chang 7 shale, geochemical data for the Chang 7 shale from Zhang et al.^85^. (**a**) Major elements, (**b**) trace elements, EF element X *=* (X_sample_/Al_sample_)/(X_background_/Al_background_), post-Archaean Australian shale (PAAS) reported by Taylor and McLennan^86^, (**c**) rare earth element, chondrite geochemical data from Zolensky et al.^87^.

Before the experiment, the geochemical characteristics of the major and trace elements in the samples were determined via inductively coupled plasma‒optical emission spectrometry (ICP‒OES) and laser ablation-inductively coupled plasma‒mass spectrometry (LA-ICP‒MS). The major elements were found to be rich in phosphorus (Fig. 3a), the trace elements were significantly enriched in U and Mo (Fig. 3b), and the distribution characteristics of the rare earth elements were consistent (Fig. 3c). The vitrinite reflectance (Ro​) of the polished block sample was determined to be 0.56%. The LECO CS-230 element analyzer showed that the total organic carbon (TOC) content was 14.7%, and the total sulfur (TS) content was 5.41%. The Rock-Eval 6 pyrolysis analysis results indicate that the pyrolysis peak temperature (T_max_) of the shale sample is 435 °C, suggesting a low level of maturity, which is consistent with the Ro​ results. The hydrocarbon generation potential (S_1_ + S_2_) was 71.4 mg/g, and the hydrogen index (HI) was 486 mg/g·TOC. In summary, the sample exhibits low maturity and a high abundance of organic matter.

The mineral and clay compositions of the shale sample were analyzed using the Philips PW-1830 Cu-Kα X-ray diffractometer. The results revealed that the clay minerals make up 55.2% of the sample, quartz 24.9%, pyrite 14.1%, and feldspar 5.8%. Among the clay minerals, illite-smectite mixed layers are the most predominant, constituting 54% of the clay minerals, followed by illite at 41%, chlorite at 3%, and kaolinite at 2%. All the above analyses were completed at the Beijing Research Institute of Uranium Geology.

### Pyrolysis apparatus and procedure

**Fig. 4:**
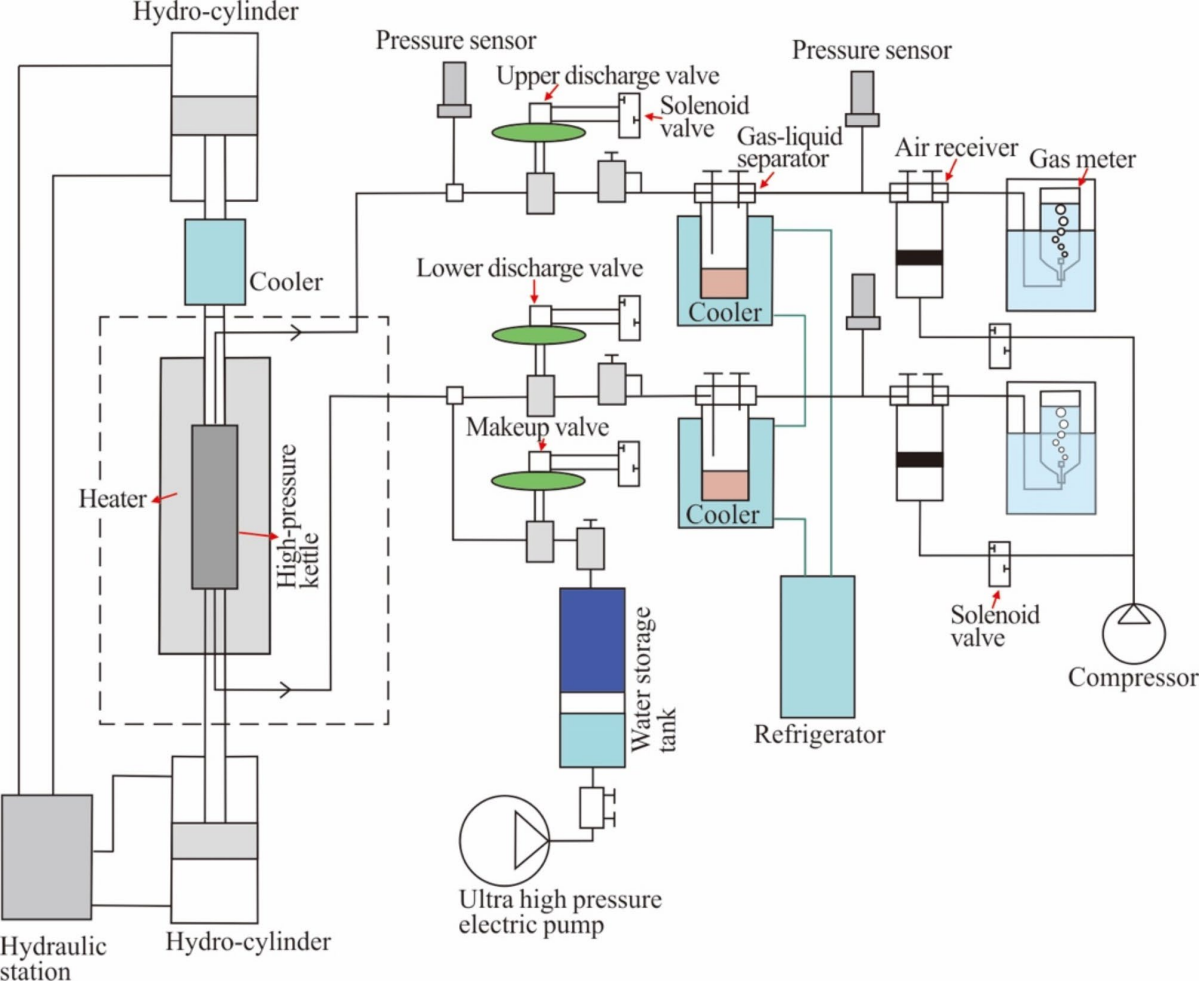
Schematic diagram of the pyrolysis system.

The pyrolysis experiment was conducted at the China Petroleum Exploration and Development Research Institute (Beijing). The experimental system consisted of a pyrolysis device and a product collection device (Fig. 4). Before the experiment, 40 g of the sample was added to 4 g of deoxygenated deionized water and placed in a 3.8 cm diameter alloy reactor. The reactor body was composed of elements such as Fe, Mn, Cr, Ti, Nb, C, Si, P, and S.

A metal filter, copper washer, and graphite ring were placed at both ends of the sample in the reactor. After loading the sample, the vertical pressure was set to 10 MPa by controlling the hydraulic device to compact the graphite ring and sealing washer of the sample in the reactor; then, the system was flushed with nitrogen gas to replace the air in the reactor. Afterwards, the vertical static pressure was set to 70 MPa, the discharge valve was closed, and a sealed pump was used to apply 20 MPa of nitrogen to the system for leakage testing. When the pressure could be maintained for at least 30 min, the system was considered airtight. In this study, the experimental temperature was a single variable, the vertical pressure was set at 70 MPa, and the pore fluid pressure was set at 30 MPa (provided by deoxidized deionized water at temperatures below 140 ℃ and provided by water vapour at temperatures above 140 ℃) to simulate the pressure conditions at a depth of 3000 m in the basin^42^. After the leakage test was completed, vacuum was applied to the gas–liquid separator and collection system, the system was flushed with nitrogen gas three times, and the airtightness was checked with a vacuum gauge.

There are generally three methods for programmed heating: isothermal pyrolysis with different holding times^43,44^, nonisothermal pyrolysis at different heating rates^45,46^, and nonisothermal pyrolysis with constant temperature intervals and heating rates^47,48^. Among them, nonisothermal pyrolysis at different heating rates is usually used to determine hydrocarbon generation kinetics. Given that this experiment focused on studying the migration characteristics of elements during different stages of shale maturation, both the composition of the pore fluid and the yield and composition characteristics of the liquid products play important roles in controlling the solubility of elements. Therefore, nonisothermal pyrolysis with a constant heating rate of 20 ℃/h was used to heat the system to different target temperatures of 60, 80, 100, 140, 180, 230, 280, 330, 350, and 370 ℃, at which point the system was maintained for 72 h. The temperature interval at the low-temperature stage was set densely to determine the effect of temperature on the element solubility. The maturity stage of the organic matter was determined from the Ro concentration in the solid residual samples. In the low-temperature stage (< 230 ℃), Ro was determined by the covariant relationship between the different heating stages of the coal rock type source rock samples in the Ordos Basin and their measured Ro^49^ due to the weak effect of Ro with increasing heating temperature during this stage.

During heating, the episodic hydrocarbon discharge pressure was set to 30 + 3 MPa; that is, when the fluid pressure in the reactor was greater than 33 MPa, the system automatically opened the valve until the fluid pressure reached 30 MPa. The product discharged from the reactor was stored in the gas-liquid separator. The experimental device used in this study simulated hydrocarbon generation and release simultaneously without stopping the heating process.

### Pyrolysis product quantification

Pyrolysis products include discharged gas, expelled liquid (oil and water), and retained oil that has not been drained in shale residue^50^. After the experiments at 140 ℃ and below, the hydrocarbon discharge valve was directly opened to remove the products from the reactor, and all the pyrolysis gas entered the gas-taking device. The pressure was recorded after the pressure gauge reached equilibrium, and the gas production was calculated by using the ideal gas state equation PV = nRT (P is the pressure (Pa), V is the gas volume (m^2^), T is the temperature (K), n is the amount of substance in the gas (mol), and R is the molar gas constant (J/mol.K). For the experiment at a temperature higher than 140 ℃, the temperature of the system was decreased to 150 ℃ before the hydrocarbon discharge valve was opened and the products in the reactor were drained. The pyrolysis gas first entered the gas‒liquid separator, which was connected to the vacuumed gas quantitative sampling pipe. After cooling by a chiller, the air outlet of the gas–liquid separator was opened to start venting and discharge the gas to push the saturated brine to the water tank. After balancing, the liquid level of the vent bottle was aligned with the scale of the gas quantitative sampling pipe, and the scale of the gas quantitative sampling pipe was read.

The oil and water mixture released from the gas‒liquid separator and the condenser was measured by a cylinder and stored in a conical flask for further analysis. The expelled oil in the reactor and piping was collected with dichloromethane and transferred to a conical flask. Dichloromethane was used for oil and water separation. The separated aqueous solution was subsequently subjected to LOA analysis. The quality of the oil was determined by the natural constant weight method. The specific procedure was as follows: The filtered oil was diluted with a mixed solution of dichloromethane to 100 ml, 50 ml was placed in a beaker for natural evaporation. The sample was weighed once every half hour, and when the mass change stabilized at ± 0.0005 mg and occurred three times continuously, the weight was considered the final effective mass of the discharged oil.

The retained oil was taken from the residue inside the reactor. Twenty grams of residue was weighed and crushed to 200 mesh. The soluble organic matter was extracted using a Thermo Fisher accelerated solvent extractor, and the extraction solvent was a mixture of dichloromethane/methanol (9:1). The retained oil was also quantified using the natural constant weight method, as described above.

### Analysis of the collected gas

The collected gas was analysed by an Agilent Wasson-7890a gas chromatograph (GC), which was equipped with three detectors: an FID for hydrocarbon gases (CH_4_, C_2_H_6_, C_2_H_4_, C_3_H_8_, C_3_H_6_, i-C_4_H_10_, n-C_4_H_10_, i-C_5_H_12_, and n-C_5_H_12_), one TCD for H_2_ and another TCD for other inorganic gases, such as CO_2_, O_2_, and N_2_. The components of the gas were quantified by means of an external standard method as follows: before the experiment, a certain volume of standard gas sample with a known molar percentage (CH_4_ = 5 mol%, CO_2_ = 5 mol%, C_2_H_6_ = 2 mol%, C_3_H_8_ = 1 mol%, N_2_ = 87 mol%) was injected into the cylinder that had previously been evacuated, which was calibrated by gas chromatography. Therefore, the volume of the gas phase product was calculated according to the gas chromatographic response between the gas phase product and the standard gas phase.

### Analysis of organic acids in discharged water

The generation of LOAs from sedimentary organic matter during maturation has been reported and investigated mostly by means of isothermal electrophoresis^25,51^, ion chromatography^52^, gas chromatography and high-performance liquid chromatography (HPLC)^53^. Among these methods, HPLC has considerable advantages, such as high sensitivity, wide detection range, and convenient pretreatment operation. Therefore, in this study, HPLC was used to analyse the organic acid composition of liquid water products (≤ 230 °C) and aqueous solutions after oil–water separation (≥ 280 °C).

A Thermo Ultima 3000 high-performance liquid chromatograph was used with an ultimate AQ-C18 column (5 μm × 4.6 mm × 250 mm; detection wavelength: 210 nm; mobile phase A: methanol; B: 0.02 M potassium dihydrogen phosphate; and gradient elution: 0–30 min = 100% B, 31–50 min = 20% A + 80% B, and 51–60 min = 100% B.) The flow rate was 0.5 ml/min, the injection volume was 10 µl, and the column temperature was 30 °C. Eighteen mixed acid reference standards, including formic acid, acetic acid, propionic acid, n-butyric acid, oxalic acid, lactic acid, citric acid, and quinic acid, were weighed, dissolved in first-class water to the corresponding concentrations and diluted to obtain reference solutions of different concentrations. After filtration through a 0.45 μm microporous membrane, the samples were directly injected to establish a standard curve of concentration and peak area.

### Analysis of solid residue

By comparing the trace element contents of residual samples with those of original samples, the migration rates of elements were determined. To investigate the migration characteristics of elements during the simulation of hydrocarbon generation and expulsion, the elemental composition of the solid residues from each experiment in this study was measured by multiple instruments. The major elements were analysed using ICP‒OES, and the loss on ignition (LOI) was determined by using ~ 1 g of sample powder at 980 °C in a muffle furnace for 30 min. Trace elements were analysed using an Agilent 7500a LA-ICP‒MS instrument, with one parallel per sample. In addition, the TOC and TS contents of the initial and residual samples were determined using a Leco CS-230 determinator. The above tests were completed at the Beijing Research Institute of Uranium Geology. The residue samples were crushed to 200 mesh and dried before analysis.

### Uncertainty analysis and simulation experiment limitations

The measurement of uncertainty is determined by the overall standard uncertainty. According to 11 groups and 2 analysis results of each group, the specific calculation is as follows:

$$\bar{x}_{i} = \frac{{x_{{i1}} \pm x_{{i2}} }}{2}$$

$$s_{i} = \sqrt {\left( {x_{{i1}} - \bar{x}_{i} } \right)^{2} + \left( {x_{{i2}} - \bar{x}_{i} } \right)^{2} }$$

$$\:{S}_{overall}^{2}=\frac{{\sum}_{i=1}^{11}\left({s}_{i}^{2}\right)}{2}$$

$$\:{u}_{overall}=\frac{{S}_{overall}}{\sqrt{11}}$$

Where, $\bar{x}_{i}$ is the average within each group, s_i_ is the variance within each group, S_overall_ is the overall standard deviation, and u_overall_ is the overall standard uncertainty. See Table 1 for overall standard uncertainty.

**Table 1:** Overall standard uncertainty of trace element analysis (ppm).

Elements	Li	Be	Sc	V	Co	Cu	Zn	Rb	Mo	Ba	Pb	Th	U	Zr
S_overall_	0.36	0.05	0.14	0.60	0.12	0.61	0.48	0.63	0.34	1.47	0.30	0.11	0.17	0.47

The hydrocarbon generation simulation experiments based on temperature-time compensation effectively reflect the characteristics of hydrocarbon products from the thermal pyrolysis of source rocks. However, under relatively high-temperature conditions, the solubility of elements in the solution is significantly affected, and this effect is difficult to quantify with the existing experimental setup.

## Results and discussion

### Hydrocarbon yields

**Fig. 5:**
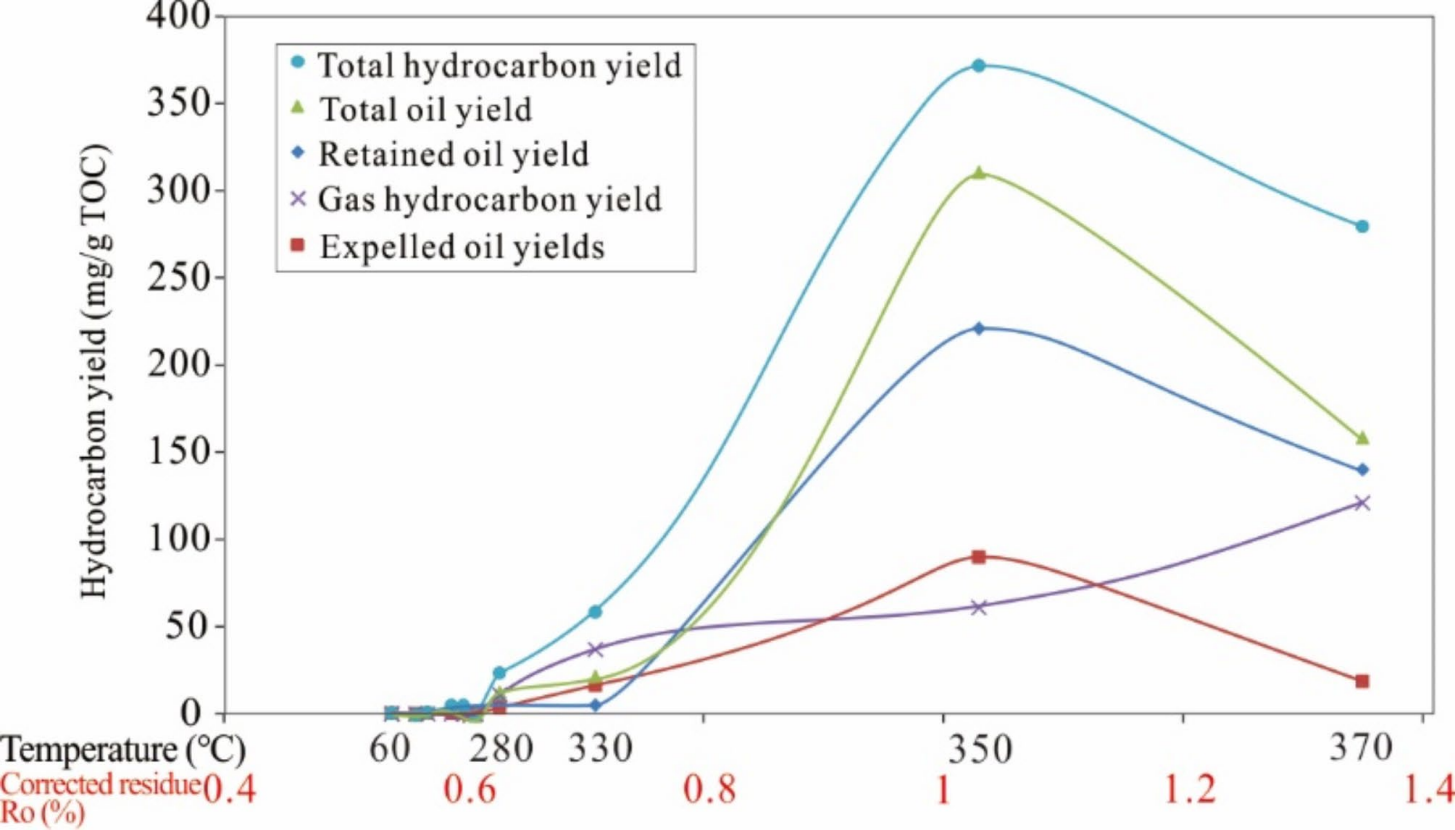
Yields of hydrocarbons with increasing final pyrolysis temperature.

The yield of hydrocarbons was determined based on the mass of both released and retained hydrocarbons, and the data are summarized in Table 2. The total oil yield = retained oil + expelled oil, and the total hydrocarbon yield = total oil yield + gaseous hydrocarbon yield. The hydrocarbon yields at 10 different heating temperatures are shown in Fig. 5; Table 2. The overall oil yield distribution exhibited unimodal characteristics, and the trends in the retained oil and expelled oil yields were similar. In the early thermal stage (60–230 °C), a small amount of liquid hydrocarbons were produced. As the temperature increased, a considerable amount of liquid hydrocarbons ere generated starting at 280 °C, and the content rapidly increased, reaching a maximum oil generation yield of 310 mg/g·TOC at 350 °C. Afterwards, the oil yield decreased significantly. Figure 5 shows that the gas hydrocarbon yield gradually increased above 280 °C and reached a yield of 121 mg/g TOC at 370 °C, but the gas hydrocarbon yield exhibited enormous growth potential with further increases in heating temperature (Fig. 5). Due to the relatively low experimental temperature in this study, the hydrocarbon products were mainly liquid hydrocarbons, and a maximum yield of gas hydrocarbons was not achieved. However, the total hydrocarbon yield gradually decreased above 350 °C, similar to the results obtained with many semiopen pyrolysis systems for terrestrial shale hydrocarbon generation^54–56^, indicating that the processes of kerogen pyrolysis and oil generation included two processes: evaporation and secondary cracking^57,58^. This study mainly involved the former.

**Table 2:** Yields (mg/g·TOC) of expelled oil, retained oil, total oil, gas hydrocarbons, and total hydrocarbons with increasing pyrolysis temperature.

Temperature (℃)	60	80	100	140	180	230	280	330	350	370
Corrected residue Ro (%)	0.54	0.56	0.57	0.59	0.6	0.61	0.63	0.71	1.03	1.35
Expelled oil	0.00	0.00	0.00	0.00	0.00	0.00	3.08	16.25	89.61	18.33
Retained oil	0.22	0.66	0.68	4.57	4.58	1.35	9.11	4.89	220.80	139.94
Total oil	0.22	0.66	0.68	4.57	4.58	1.35	12.19	21.14	310.41	158.27
Gas hydrocarbon	0.00	0.00	0.00	0.00	0.00	0.00	11.00	37.00	61.00	121.00
Total hydrocarbon	0.22	0.66	0.68	4.57	4.58	1.35	23.19	58.14	371.41	279.27

### Yields of organic acid

**Fig. 6:**
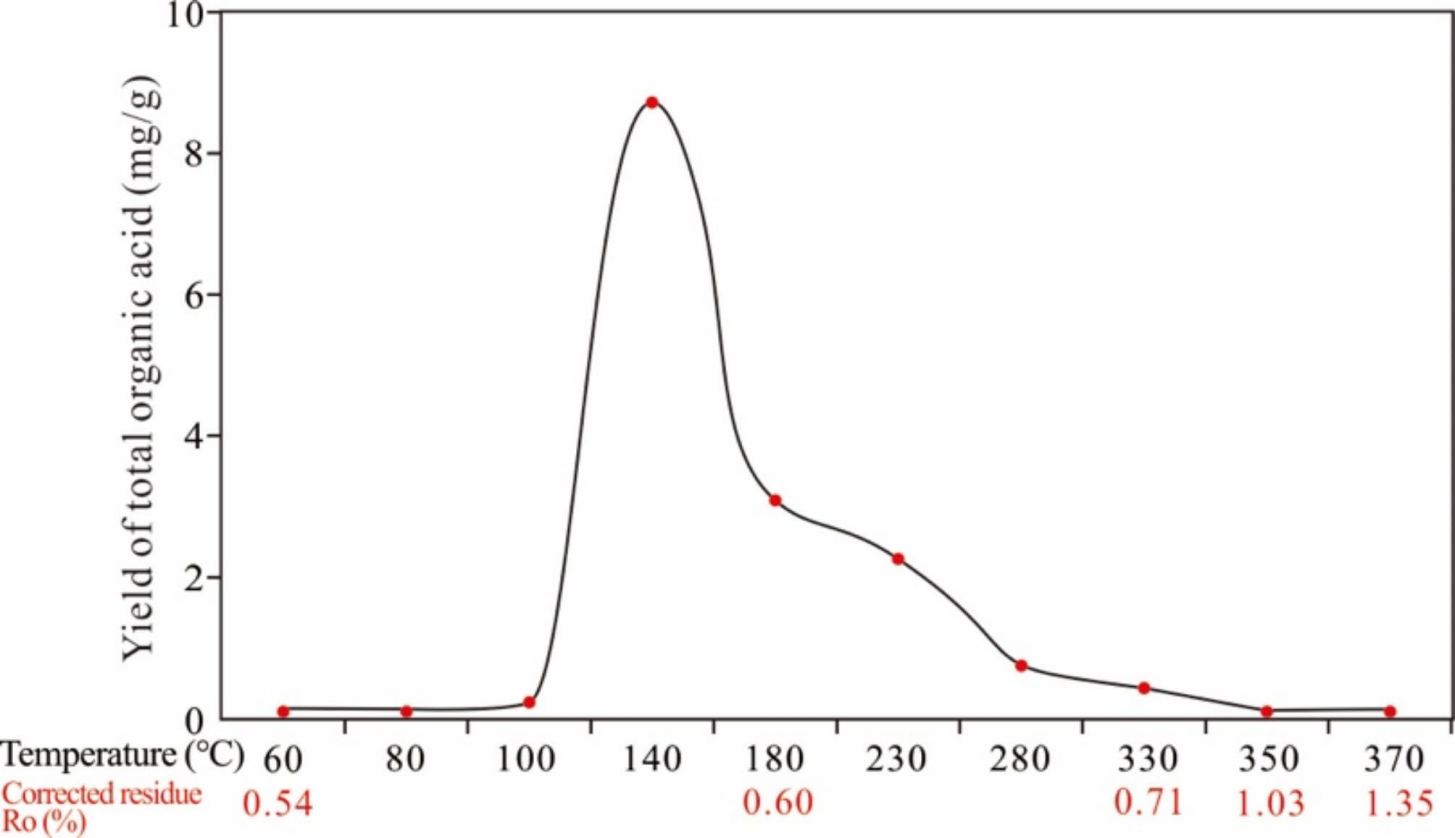
Yields of total acids with increasing final pyrolysis temperature.

Figure 6 shows the trend of the total yield of LOAs. Few LOAs were produced below 100 ℃, but this yield rapidly increased from 100 to 140 ℃ and reached a maximum of 8.58 mg/g·TOC at 140 ℃; subsequently, as the temperature increased, the yield decreased and approached zero when the temperature reached 350 ℃, indicating that organic acids were mainly produced before hydrocarbon generation, similar to the findings of Surdam et al.^59^. The discharge water production at different stages ranged from 9.5 to 11.3 ml, with corresponding total acid concentrations ranging from 55.1 to 5046.4 mg/L (Table 3), and the maximum concentration occurring at 140 °C, which is comparable to the maximum value of some oilfield reservoir water (approximately 5000 mg/L)^60^.

**Table 3:** Organic acid concentrations (mg/L) in discharged water and total yield.

Temperature (℃)	60	80	100	140	180	230	280	330	350	370
Total acids concentration	121.3	74.6	795.9	5058.1	1747.6	1262.4	353.3	327.6	216.5	69.1
Discharge water (mL)	11.2	12.6	10.5	10.1	10.3	9.8	10.5	9	10.5	9.6
Acid yield (mg/g·TOC)	0.23	0.16	1.42	8.69	3.06	2.10	0.61	0.46	0.35	0.11

### Migration rate of trace elements

**Fig. 7:**
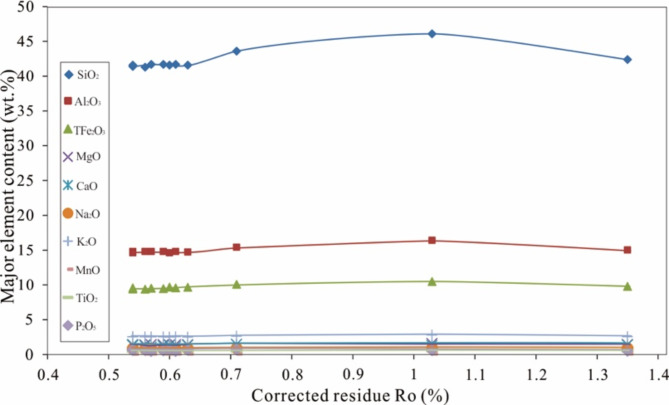
The major element contents in the residual samples with increasing final pyrolysis temperature.

The major elements in the solid residue after pyrolysis were analysed, and the results are shown in Supplementary Table S1. Apparently, the major elements rarely dissolved and migrated during pyrolysis. As the pyrolysis temperature increased, a large amount of kerogen transformed into hydrocarbons, and the major elements became more enriched (not excluding the influence of loss on ignition) (Fig. 7).

**Fig. 8:**
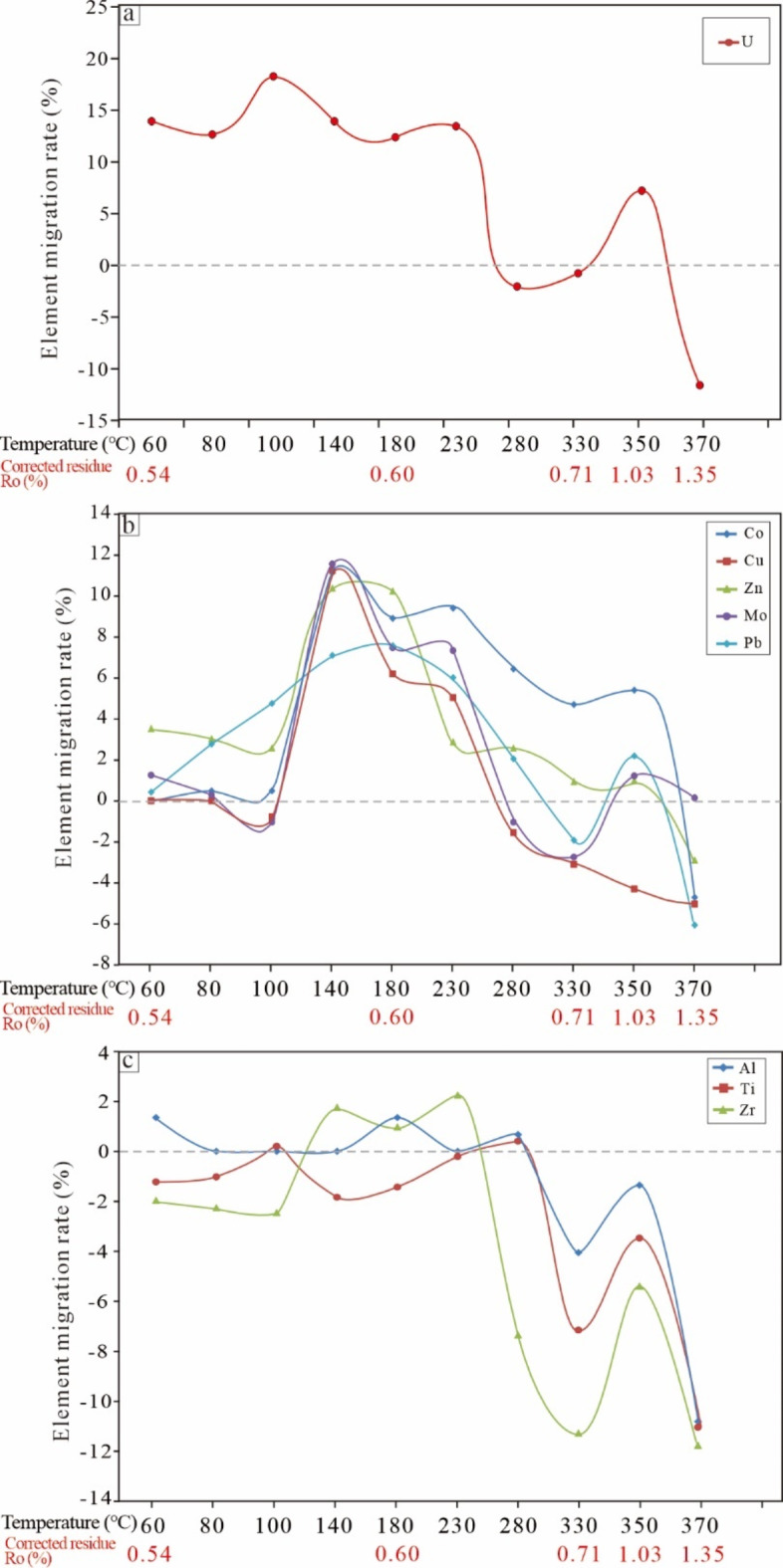
Trace element leaching rate of residual samples with increasing final pyrolysis temperature: (**a**) U, (**b**) Cu, Pb, Zn, Co, and Mo, and (**c**) Al, Ti and Zr.

The trace element content of the residual sample was compared with that of the original sample (Supplementary Table S2), and the trace element migration rate diagrams are displayed in Fig. 8. The trace elements followed different migration paths (Fig. 8). During the low-temperature stage (60–100 °C), as the temperature increased, the solubility of uranium in water increased, and the migration of uranium gradually increased, reaching the highest leaching rate of 18.8% (Fig. 8a). During the acid production stage before hydrocarbon generation at 140–230 °C, the leaching rate of uranium remained high and fluctuated between 12.1% and 13.9%. Subsequently, during the early oil generation stage at 280–330 °C, the leaching rate of uranium rapidly decreased to zero; a negative leaching rate indicated that the element mobility was significantly lower than the kerogen conversion rate, indicating that the element was not significantly leached. Only at the main oil generation stage at 350 °C did the leaching rate increase up to 7.2%, indicating that uranium had a relatively low migration rate during the hydrocarbon generation stage.

The leaching rates of Cu, Pb, Zn, Co, and Mo were relatively low at 60–100 °C (Fig. 8b). During the large-scale generation of LOAs at approximately 140 °C, the leaching rates of metal elements significantly increased, reaching the highest level of approximately 12% for Cu, Zn, Co, and Mo and 8% for Pb. Subsequently, during hydrocarbon generation between 280 °C and 370 °C, the leaching rates of elements significantly decreased, reaching the lowest values at 370 °C. At the maximum hydrocarbon generation stage at 350 °C, the leaching rates of Co, Zn, Mo, and Pb showed a slight increase, indicating that liquid hydrocarbons contribute to the migration of trace elements.

In addition, several elements, such as Al, Ti, and Zr, exhibited good stabilities and did not undergo significant leaching or migration throughout the thermal evolution simulation stage (Fig. 8c). Ti is a lithophilic element that often exists in terrigenous clastic rocks in the form of titanium-bearing oxides and silicates^61^ and is one of the least active metallic elements in nature. Zr, a homologue of Ti, has stable geochemical properties during sedimentary diagenesis and is often used as an indicator of terrigenous clastic input^62^; therefore, Zr can represent immobile elements during the maturation of source rocks.

### The mechanism of element migration

The sedimentation of the Yanchang Formation in the Ordos Basin was accompanied by volcanic eruptions, and hundreds of layers of tuff were interbedded with the lake basin sediments. Since uranium is a strongly incompatible element, it will continue to be enriched as magma evolves^63^. Therefore, these volcanic eruptions provided abundant uranium to the lake water during the subsequent leaching process. Generally, the composition of pore water in the sediment at the bottom of a lake is closely related to the water in the lake, and the mineralization degree of the pore water in the bottom layer of the sediment is greater than that in the surface layer; that is, the thicker the sediment is, the greater the mineralization degree of the pore water^64,65^. Uranium is a variable-valent element that is sensitive to oxygen fugacity. In oxygen-rich surface water bodies, UO_2_^2+^ uranyl ions with high solubility are present, and in oxygen-deficient to reducing environments, they transform into UO_2_, with significant decreases in solubility and precipitation. Free UO_2_^2+^ in lake water is continuously extracted by bottom sediments, enters pore water, and is reduced in low Eh sedimentary environments, adsorbing on organic matter, clay minerals, and collophanite^37,66^.

During the early stages of compaction and diagenesis, as burial depth increases, the porosity gradually decreases, accompanied by the discharge of a large amount of pore water saturated with uranium. But the thermodynamic calculations have shown that under reduced oxygen fugacity conditions, the solubility of uranium (U^4+^) in solution (SO_4_^2−^, Cl^−^, CO_3_^2−^, HPO_4_^2−^) is only a few ppb^67–69^. However, these studies define the transport environment of uranium as an inorganic system. In the basin, the role of sedimentary organic matter is not limited to the provision of reducing agents, and the presence of acidic organic components such as humic acid and organic acids affects the dissolution and migration of uranium^4^. Li et al.^70^ studied the solubility of UO_2_^2+^ and U^4+^ ions in humic acid solutions through dialysis and ultrafiltration techniques and estimated the equilibrium constants K of different types of humic acids on UO_2_^2+^ and U^4+^ ions. The results showed that the magnitudes of the equilibrium constants of humic acids on UO_2_^2+^ and U^4+^ ions were almost the same (Table 4), indicating that organic fluids have the potential to become uranium ore-forming fluids in reducing environments. This study detected a high content of organic acids (especially quinic acid) through HPLC, which may be the product of thermal decomposition of humic acid^71,72^. It is believed that during the diagenetic process of uranium-rich shale, some uranium migrated with the discharge of pore water under the action of organic acids and humic acids, which is also a potential factor leading to the existence of some highly radioactive anomalous sandstones in the upper part of the Yanchang Formation uranium-rich shale^73^.

As the burial depth (temperature) increases, the activity of pore fluids decreases, and the leaching rate of uranium decreases. In the early stages of oil generation at 280 ℃ and 330 ℃, uranium does not migrate significantly. However, during the peak stage of oil generation at 350 ℃, as a large amount of kerogen is converted into liquid hydrocarbons, the leaching rate of uranium increases again, indicating that uranium molecules can significantly migrate with hydrocarbon-containing fluids during the hydrocarbon generation and expulsion process of source rocks. In addition, by measuring the elemental concentrations of oil products at 350 ℃, the results showed that their uranium content was 187 ppb (Supplementary Table S2), which is 57 times greater than that of surface water bodies (seawater 3.3 ppb^74^). However, there have been no reports of abnormal crude oil radioactivity in the oil fields in the southern part of the basin. Therefore, it is believed that the U leaches and migrates with the hydrocarbons generated from uranium-rich shale, and is not stored in crude oil but tends to remain in oil-related oilfield water.

The leaching rate of metal elements such as Cu, Pb, Zn, Mo, and Co is strongly coupled with the yield of organic acids, indicating that the organic acids generated by kerogen pyrolysis can significantly promote the dissolution and migration of some trace metal elements in shale. The electrostatic adsorption generated by organic acids in solution is due to the aggregation of carboxyl ions, which generates electrostatic attraction on metal ions. Intermolecular/intramolecular complexation/chelation allows organic acids to bind heavy metal ions to form stable chelates^75–77^. At present, research on the complexation and migration of metal elements by organic acids is limited mainly to the epigenetic environment. In addition to dissolving sandstone reservoirs, deep source rocks in basins can also produce large amounts of organic acids, which are also solvents for trace metal elements.

Additionally, tectonic activity is a potential factor influencing the migration patterns of elements, primarily by controlling the porosity of shale and the thermal evolution process. As burial depth (temperature) increases, shale porosity decreases, leading to reduced pore fluid mobility. This trend exhibits an exponential function characteristic: at shallower burial depths (e.g., less than 500 m), porosity decreases sharply with increasing depth. When the burial depth is greater (e.g., more than 3000 m), the change in porosity with depth becomes significantly slower^78,79^. Therefore, it can be inferred that the basin’s tectonic subsidence rate significantly affects the migration patterns of elements in shale. At a certain burial depth, an appropriate subsidence rate will favor the leaching of elements in the shale. For example, when the shale is at a relatively shallow depth (500–1000 m) with slow tectonic subsidence, the shale has a higher pore fluid content, which is conducive to the generation of large amounts of low molecular weight organic acids (LOAs), thereby promoting the leaching of elements.

**Table 4:** Stability constants for uranium-organic complexes^70^.

Uranium ion	Ligand	K_i_	*n*_i_ (mmol/g)
UO_2_^2+^	HA	5.4 × 10^6^	1.0
		5.3 × 10^4^	9.5
	FA	2.7 × 10^7^	0.2
		3.6 × 10^5^	3.8
	TA	2.3 × 10^6^	0.6
		9.2 × 10^4^	11.4
U^4+^	HA	9.5 × 10^6^	0.5
		3.2 × 10^4^	4.5
	FA	4.4 × 10^6^	0.3
		8.8 × 10^4^	1.8
	TA	8.5 × 10^6^	0.9
		1.1 × 10^5^	4.5

HA: humic acid, FA: fulvic acid, TA: tannic acid, K_i_: stability constant, n_i_: the number of binding sites per gram of organic ligand.

### Geochemical data statistics of source rocks in the Ordos Basin

Experiments have shown that uranium in shale can significantly leach and migrate during hydrocarbon generation. However, under real geological conditions, key evidence is still needed to determine whether uranium in shale has migrated. To this end, this study summarizes the published geochemical data of the Chang 7 member source rocks in the Yanchang Formation of the Ordos Basin, including 406 samples from 16 articles (Supplementary Table S3). Given that most sample data do not include maturity data such as Ro and T_max_, the sample statistical results are divided according to the current burial depth of the source rocks, which is divided into 0–1 km, 1–2 km, and 2–3 km. The main basis for this division is that the overall tectonic activity of the basin is relatively complete; that is, the strata in the southern and northern parts of the basin have undergone similar subsidence and uplift processes since the Triassic period^80,81^. Based on this, it is believed that the current burial depth of the Yanchang Formation shale can roughly serve as a benchmark for relative maturity.

**Fig. 9:**
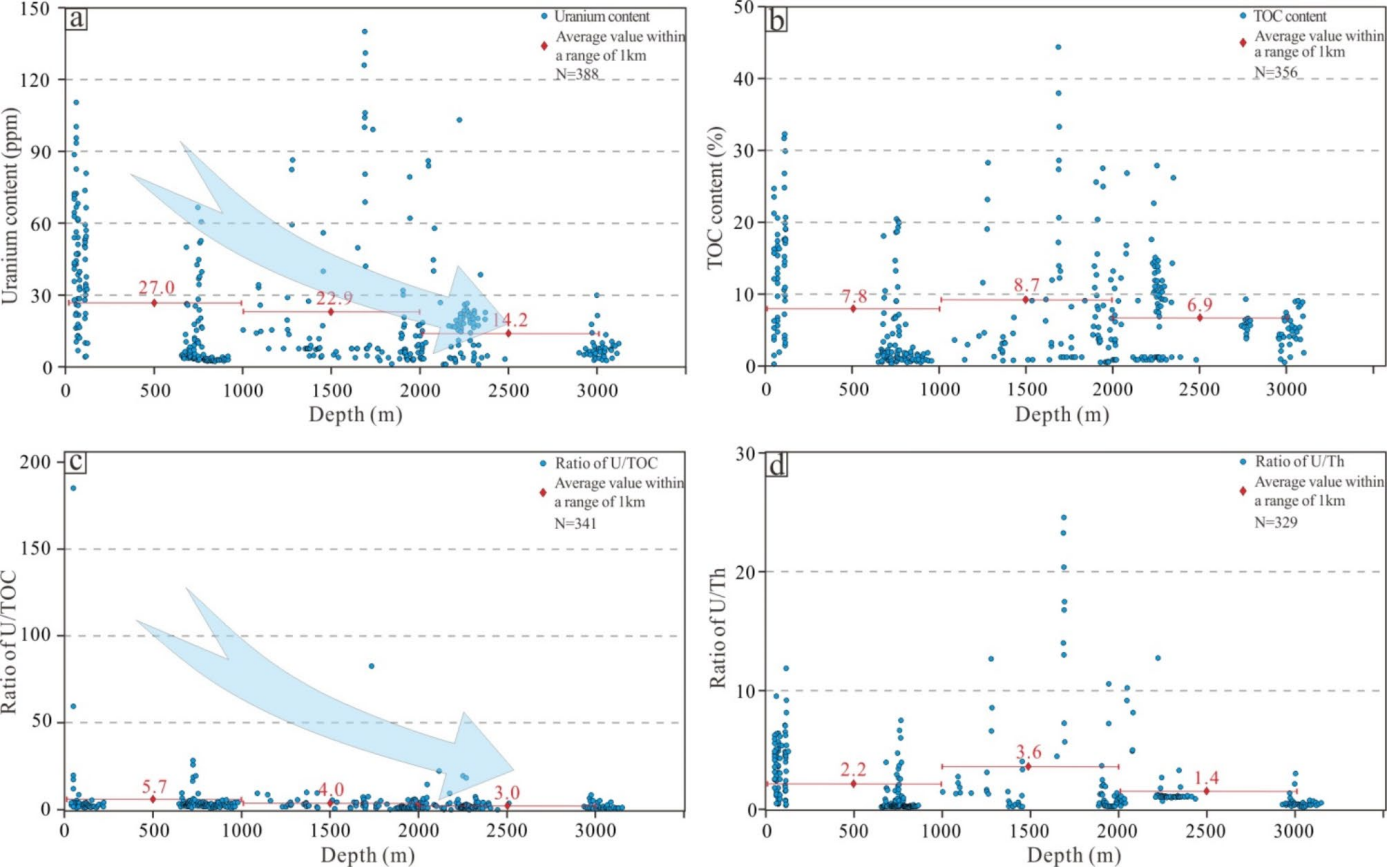
Geochemical characteristics of black shale in the Chang 7 member of the Yanchang Formation at different burial depths in the Ordos Basin: (**a**) U content, (**b**) TOC content, (**c**) U/TOC ratio, and (**d**) U/Th ratio.

**Fig. 10:**
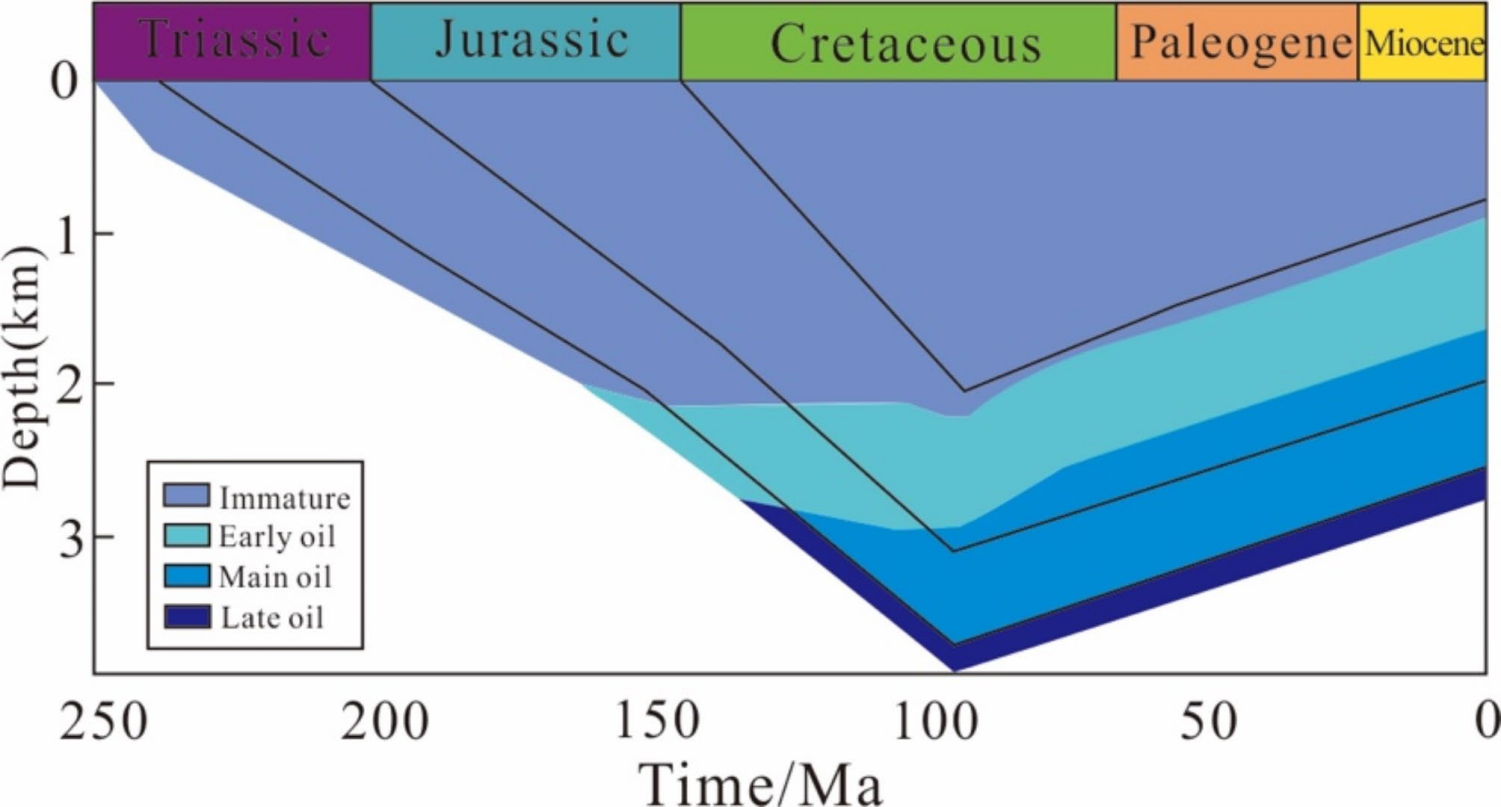
Simulation results of the buried hydrocarbon generation history in the Ordos Basin^82^.

The statistical results revealed that the uranium abundance characteristics of the Chang 7 member shale in the extended group vary significantly at the different depths (Fig. 9a). The samples with shallow burial depths of 0–1 km, especially the profile samples, have a higher overall uranium content, with an average uranium content of 27.0 ppm. As the burial depth increases, the uranium content of the shale at depths of 1–2 km decreases relatively, with an average of 22.9 ppm. At depths of 2–3 km, the uranium content continues to decrease, with an average of 14.2 ppm. The basin simulation results of Cui et al.^82^ showed that the Chang 7 source rocks entered the oil generation stage at a depth of approximately 2 km (Fig. 10). Although the relationship between the maturity of source rocks and the current burial depth is difficult to quantify due to the late uplift of the basin, the statistical results still show that the Chang 7 source rocks with burial depths greater than 2 km have relatively low organic matter abundances, with an average TOC content of 6.9%, which is significantly lower than the average TOC content of 7.8 and 8.7% for 0–1 km and 1–2 km, respectively (Fig. 9b). This indicates that the hydrocarbon conversion rate of kerogen in source rocks with a burial depth of more than 2 km is higher, and uranium were discharged with hydrocarbon substances, resulting in lower content.

It is noteworthy that simulation experiments indicate a low overall leaching rate of uranium during the hydrocarbon generation and expulsion stages. Significant leaching is only observed at the peak hydrocarbon generation stage (350 °C). However, under actual geological conditions, due to the influence of the geothermal gradient, the thermal evolution temperature of source rocks during maturation does not reach the initial hydrocarbon generation temperature (280 °C) observed in the simulation experiments. Consequently, organic acids do not degrade extensively. Therefore, the role of organic acids in hydrocarbon generation during geological history remains significant. Correspondingly, statistical results show that shales buried deeper than 2 km exhibit a significant decrease in uranium content.

Although the TOC contents of the 0–1 km and 1–2 km long source rocks did not decrease with increasing depth, the uranium content decreased significantly; that is, before the oil generation period, with increasing burial depth, the porosity of the shale decreased, and the discharge of pore fluid removed uranium, consistent with the uranium leaching phenomenon observed in the low-temperature stage (< 230 ℃) in this experiment.

An anoxic to reductive water environment is conducive to the preservation of sedimentary organic matter and uranium enrichment. Therefore, there is a good positive correlation between uranium and TOC in anoxic to reductive environment^15,83^. The statistical results show that the R^2^ of uranium and TOC is 0.54 (Supplementary Table S3). The U/TOC ratio gradually decreases with increasing depth, which may be due to the higher leaching rate of uranium than that of kerogen (Fig. 9c). Regional differences in sedimentary environments will affect the objectivity of the sample set. The U/Th ratio is a common indicator of the oxygen phase of the palaeosedimentary environment. A U/Th > 0.5 reflects an anoxic to reducing environment^84^. The data show that the average U/Th values of the samples at different depths are greater than 0.5 (Fig. 9d), indicating that the shale samples from the Chang 7 member have redox conditions similar to those of the ancient lake, but the U/Th values are relatively low due to the migration of uranium caused by hydrocarbon expulsion in source rock buried more than 2 km.

The geochemical statistical results for shale samples from the Chang 7 member at different burial depths revealed that during deposition, with the discharge of pore water and hydrocarbon-bearing fluid, the uranium in the shale also migrated. Therefore, uranium-rich shale is an important deep uranium source during exudative uranium mineralization. These results are expected to contribute to the exploration of sandstone-type uranium deposits in the basin from the redox type at the edge of the basin to the exudation type in the hinterland of the basin.

## Conclusion

To explore the migration law of uranium during the maturation of source rocks, a thermal simulation experiment of uranium-rich shale was carried out, and the elemental migration and fluid composition characteristics of shale at different thermal stages were investigated. Moreover, the migration law of uranium under natural geological conditions was summarized based on the geochemical statistical data of the Chang 7 shale in the Ordos Basin. The results showed the following:

1. Uranium can leach and migrate significantly before hydrocarbon generation (Ro < 0.61%, T ≤ 230 ℃), and the leaching rate was between 12.1% and 18.8%. However, during the hydrocarbon generation stage (0.63% < Ro < 1.35%, 280 ℃≤T ≤ 370 ℃), the leaching rate of uranium was generally low, ranging from 0 to 7.2%. The maximum leaching rate of uranium (7.2%) in this stage corresponded to the main oil generation stage (T = 350 °C, Ro = 1.03%). Cu, Pb, Zn, Mo, and Co also migrated considerably during the early stage of thermal evolution, with leaching rates ranging from 2.9 ~ 11.6%, with an average of 8.2%.
2. The results of HPLC analysis of the liquid products showed that the yield of LOAs was high in the early stage of thermal maturation of the shale, reaching a maximum yield of 8.58 mg/g TOC at 140 ℃ (Ro = 0.59%), and the LOA yield exhibited good correlation with the leaching rate of trace elements such as Cu, Pb, Zn, Co and Mo. The generation of LOAs by kerogen pyrolysis in source rocks was conducive to the leaching and migration of trace metal elements.
3. The results of geochemical analysis of the Chang 7 shale in the Ordos Basin show that the average uranium content (14.2 ppm) of the samples with large burial depths (> 2 km) is significantly lower than that of the 0 ~ 1 km (27.0 ppm) and 1 ~ 2 km (22.9 ppm) samples, indicating that with increasing burial depth, uranium can leach and migrate significantly during the stage of pore water discharge and hydrocarbon generation, which is consistent with the experimental results.

Therefore, the uranium-rich shale in the deep part of the sedimentary basin can be considered an important uranium source rock for exudative sandstone-type uranium deposits, and the uranium-bearing property of the deep source rock may be considered in the future exploration of sandstone-type uranium deposits in the hinterland of the basin. The characteristics of mineralizing fluids associated with the mineralization processes of exudative sandstone type uranium deposits may become one of the important areas of future research.

## Electronic supplementary material

Below is the link to the electronic supplementary material.

Supplementary Material 1

## Data Availability

All data generated or analyzed during this study are available in Supplementary Table.
